# Supplementary material for: The oocyte zinc transporter Slc39a10/Zip10 is a regulator of zinc sparks during fertilization in mice
Source: eLife. 2025 Dec 11;14:RP106616. doi: 10.7554/eLife.106616 (PMC12698087; doi:10.7554/eLife.106616)
Supplement: Figure 2—figure supplement 1—source data 1. [file elife-106616-fig2-figsupp1-data1.zip › Figure 2figure supplement 1_ Source Data 1.pdf]

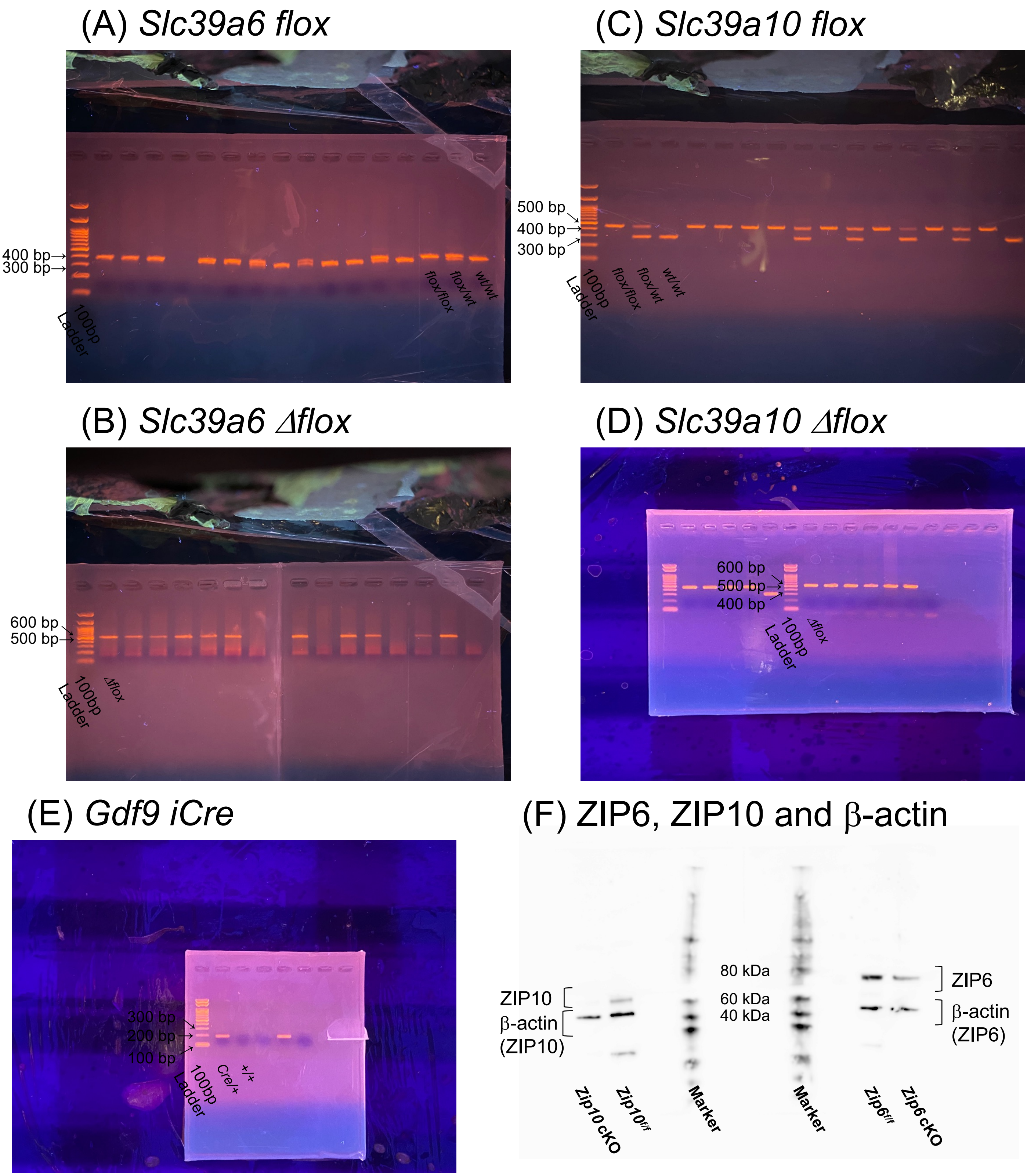

Figure 2-figure supplement 1-source data 1. Original gels corresponding to Figure 2-figure supplement 1C. Gene Ladder 100 (316-06951; NIPPON GENE) was employed as DNA size markers. (A) *Slc39a6 flox* gel used lanes 1 and 15-17 (other lanes; not shown in Figure 2-figure supplement 1C). (B) *Slc39a6 Δflox* gel used lanes 1 and 2 (other lanes; not shown in Figure 2-figure supplement 1C). (C) *Slc39a10 flox* gel used lanes 1-4 (other lanes; not shown in Figure 2-figure supplement 1C). (D) *Slc39a10 Δflox* gel used lanes 7 and 8 (other lanes; not shown in Figure 2-figure supplement 1C). (E) *Gdf9 iCre* gel used lanes 1 and 2 (other lanes; not shown in Figure 2-figure supplement 1C). All images were converted to grayscale and used. (F) Original membranes corresponding to Figure 2-figure supplement 1D. Biotinylated Protein Ladder Detection Pack (#7727; Cell Signaling Technology) were employed as molecular weight marker. After the WB blocking step, one membrane was cut vertically between molecular weight markers and further cut horizontally between 50-60 kDa. The left membrane (50-60 kDa and above) was reacted with the ZIP10 antibody, and the right membrane (below 50-60 kDa) was reacted with the β-actin antibody. The paper used the membranes with their sides reversed. Similarly, the right membrane (50-60 kDa and above) was reacted with the ZIP6 antibody, and the left membrane (below 50-60 kDa) was reacted with the β-actin antibody.
