# Supplementary figures and images for: The oocyte zinc transporter Slc39a10/Zip10 is a regulator of zinc sparks during fertilization in mice
Source: eLife. 2025 Dec 11;14:RP106616. doi: 10.7554/eLife.106616 (PMC12698087; doi:10.7554/eLife.106616)

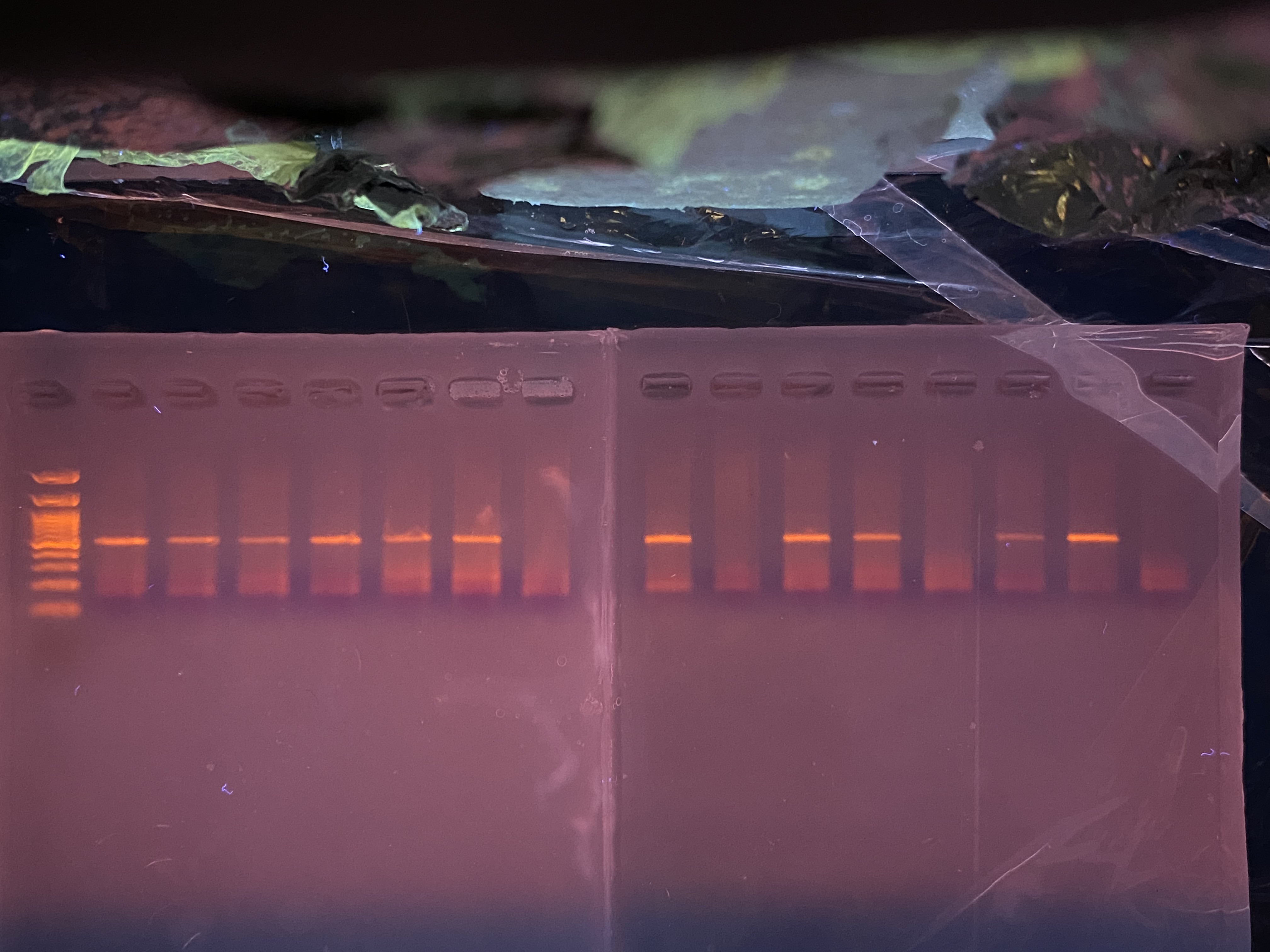

Supplement: Figure 2—figure supplement 1—source data 2. [file elife-106616-fig2-figsupp1-data2.zip › Figure 2-figure supplement 1_Source data 2/Slc39a6flox Null_Genotyping_IMG_3057.jpeg]

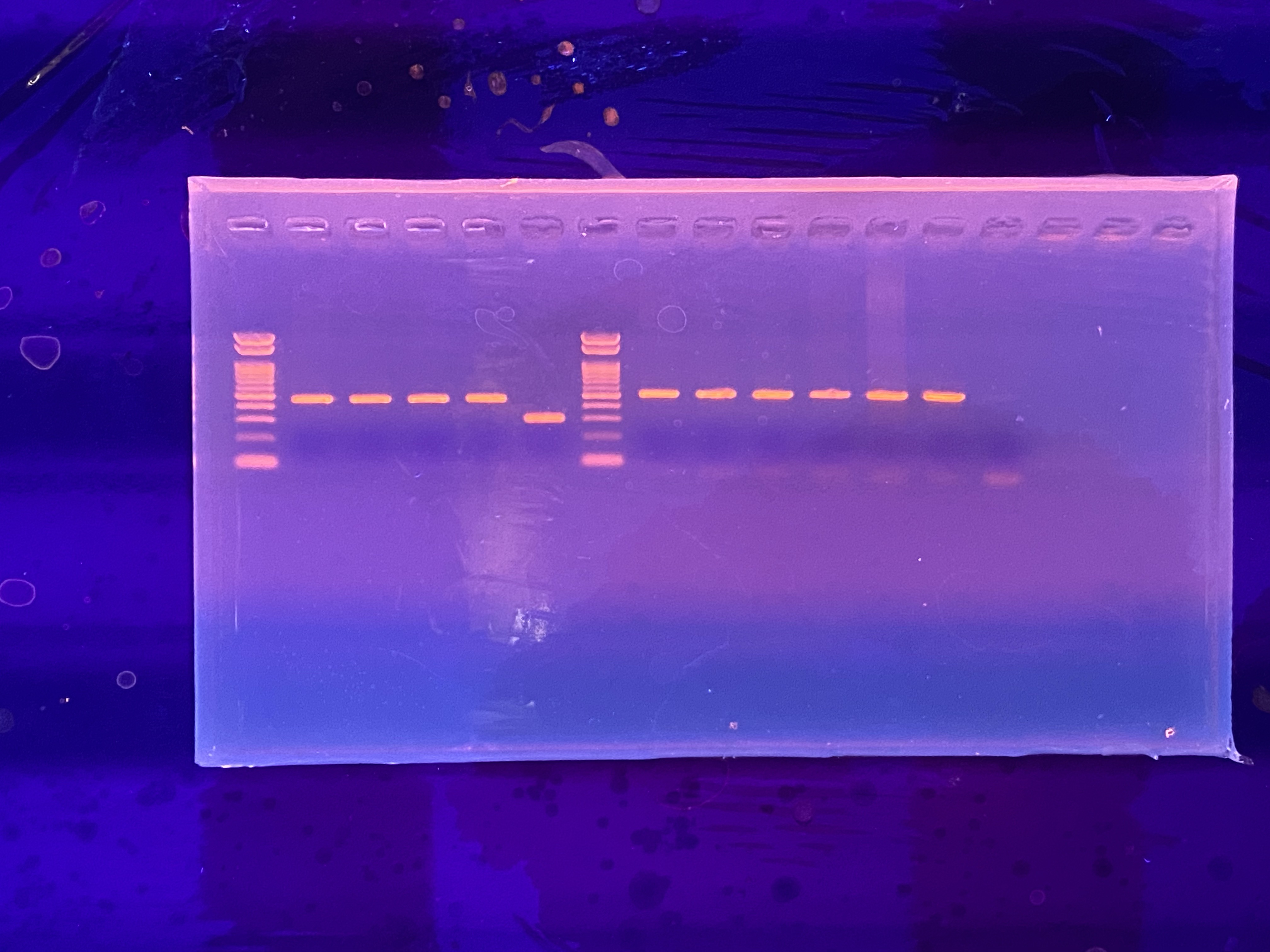

Supplement: Figure 2—figure supplement 1—source data 2. [file elife-106616-fig2-figsupp1-data2.zip › Figure 2-figure supplement 1_Source data 2/Slc39a10flox Null_Genotyping_IMG_5156.jpeg]

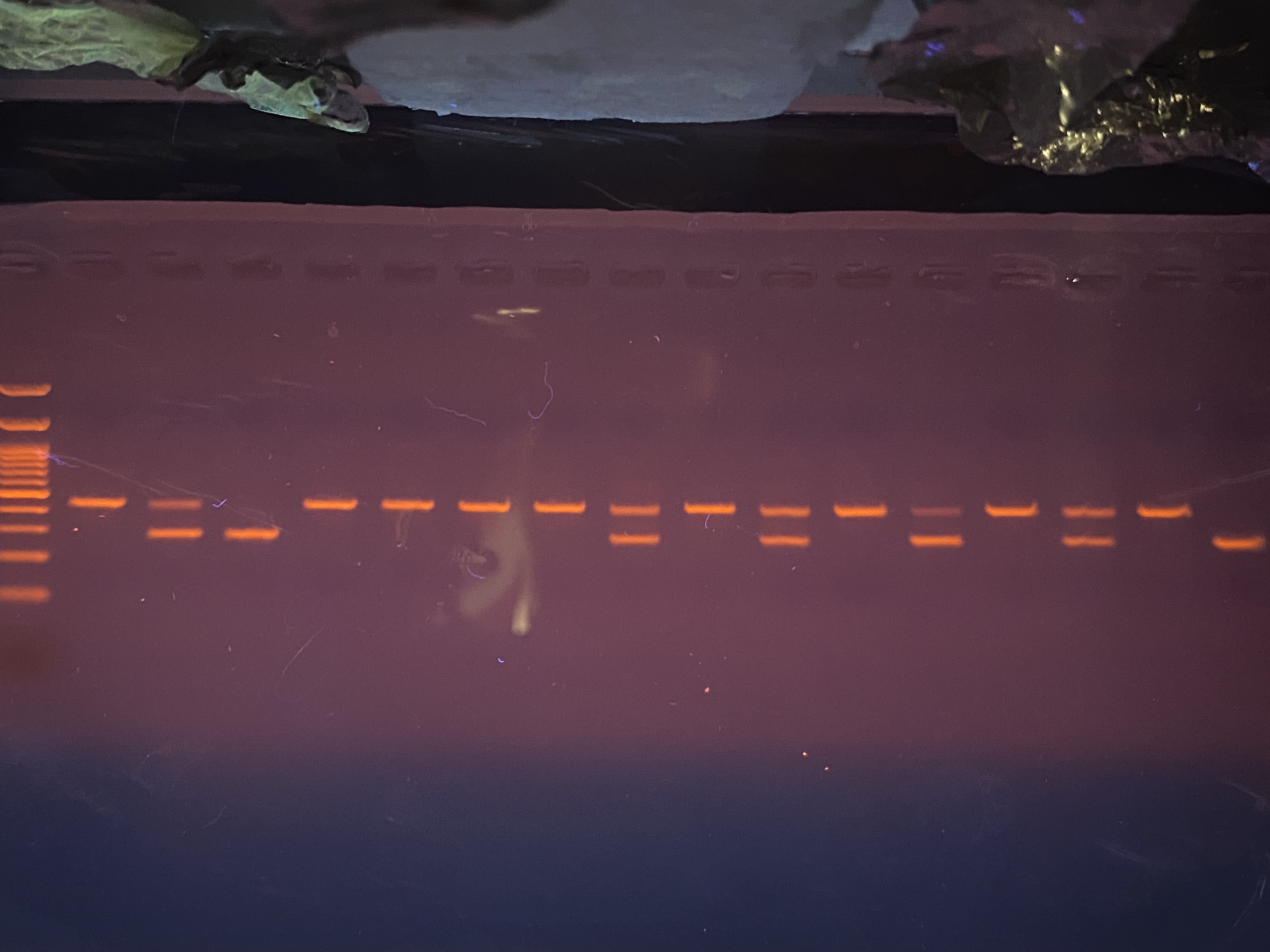

Supplement: Figure 2—figure supplement 1—source data 2. [file elife-106616-fig2-figsupp1-data2.zip › Figure 2-figure supplement 1_Source data 2/Slc39a10flox_Genotyping_IMG_3857.jpeg]

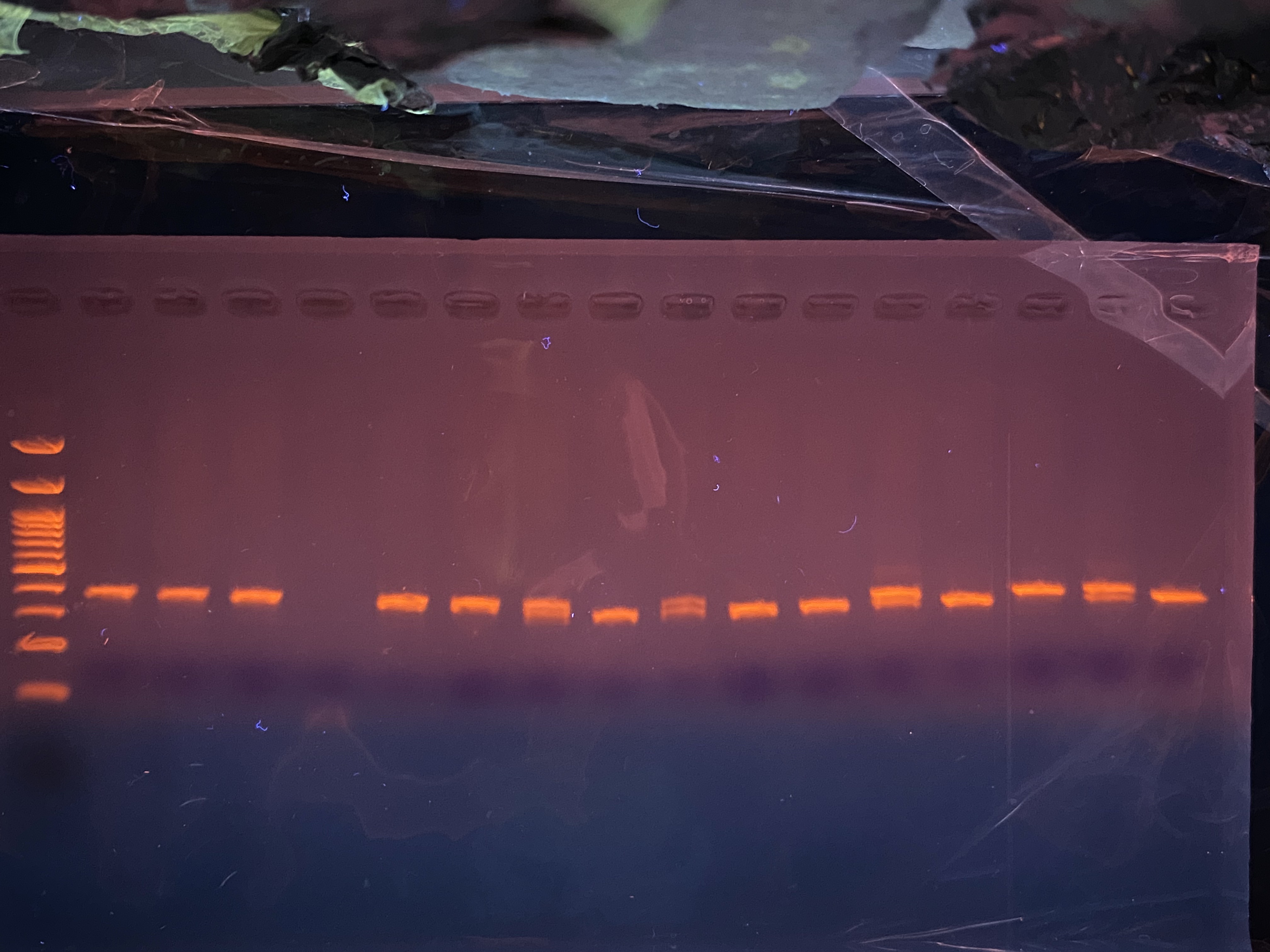

Supplement: Figure 2—figure supplement 1—source data 2. [file elife-106616-fig2-figsupp1-data2.zip › Figure 2-figure supplement 1_Source data 2/Slc39a6flox_Genotyping_IMG_3059.jpeg]

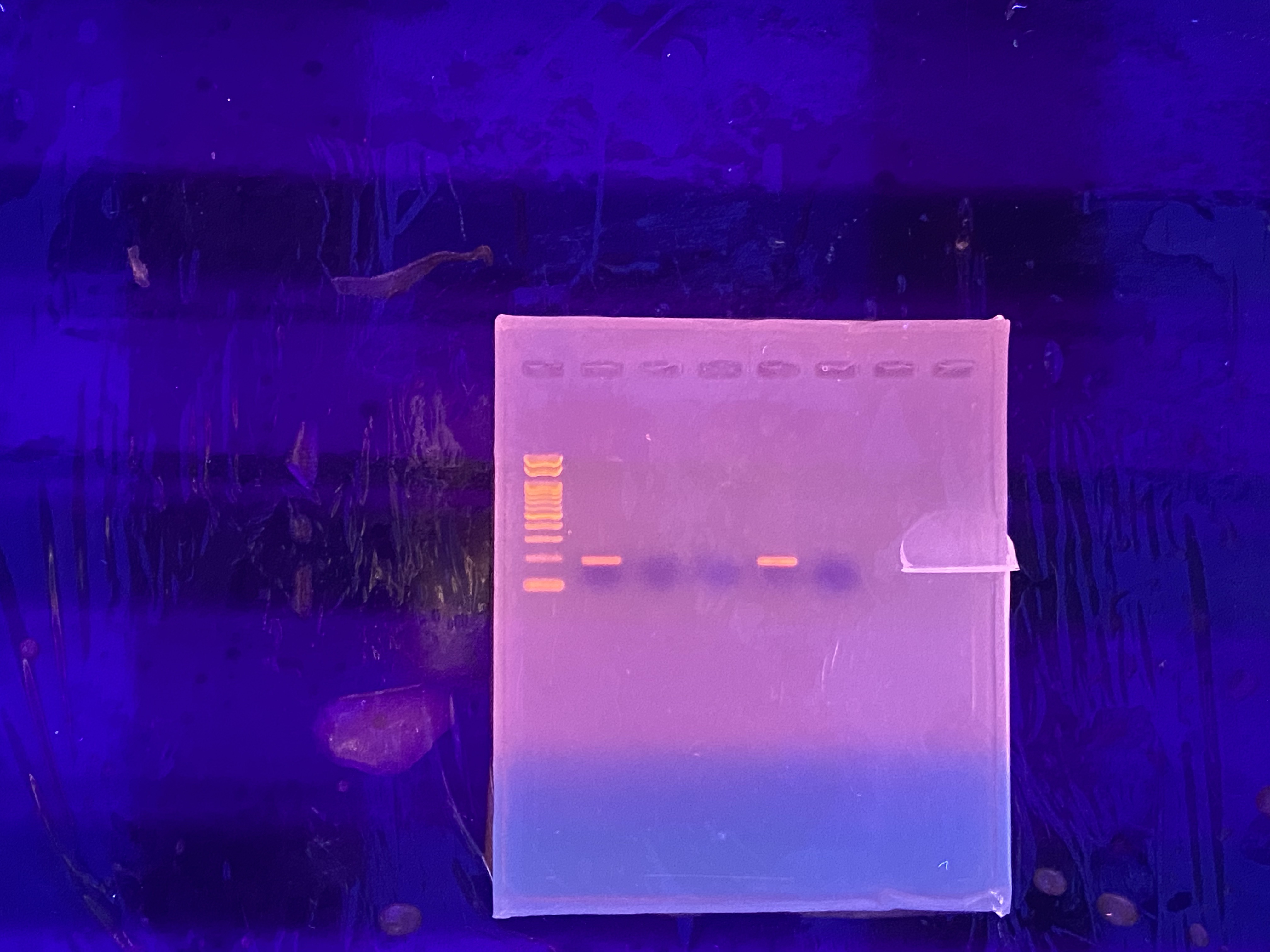

Supplement: Figure 2—figure supplement 1—source data 2. [file elife-106616-fig2-figsupp1-data2.zip › Figure 2-figure supplement 1_Source data 2/Gdf9iCre_Genotyping_IMG_5134.jpeg]

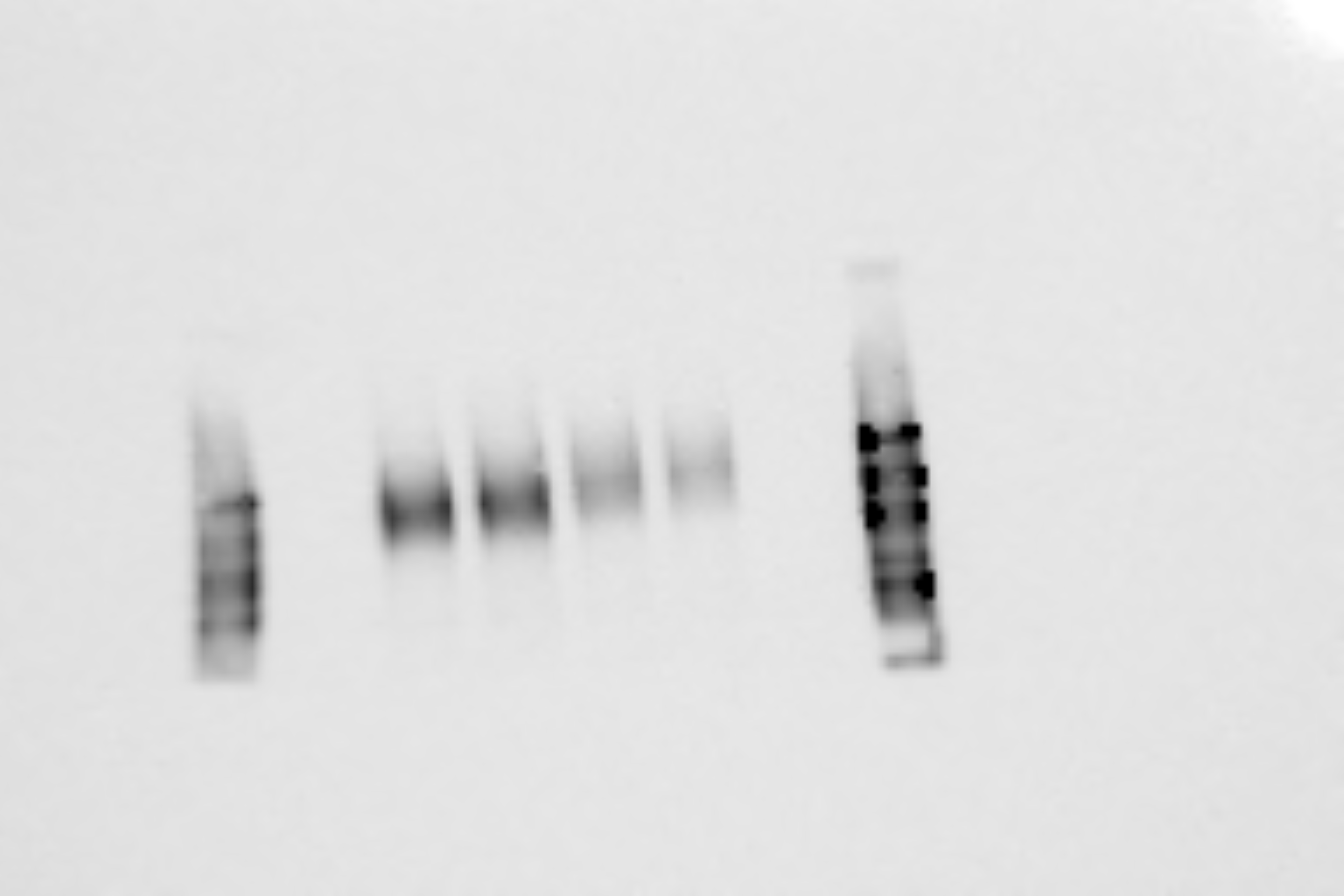

Supplement: Figure 4—source data 2. [file elife-106616-fig4-data2.zip › Figure 4_Source Data 2/ZP2 WB_20230328_1450_20.tif]

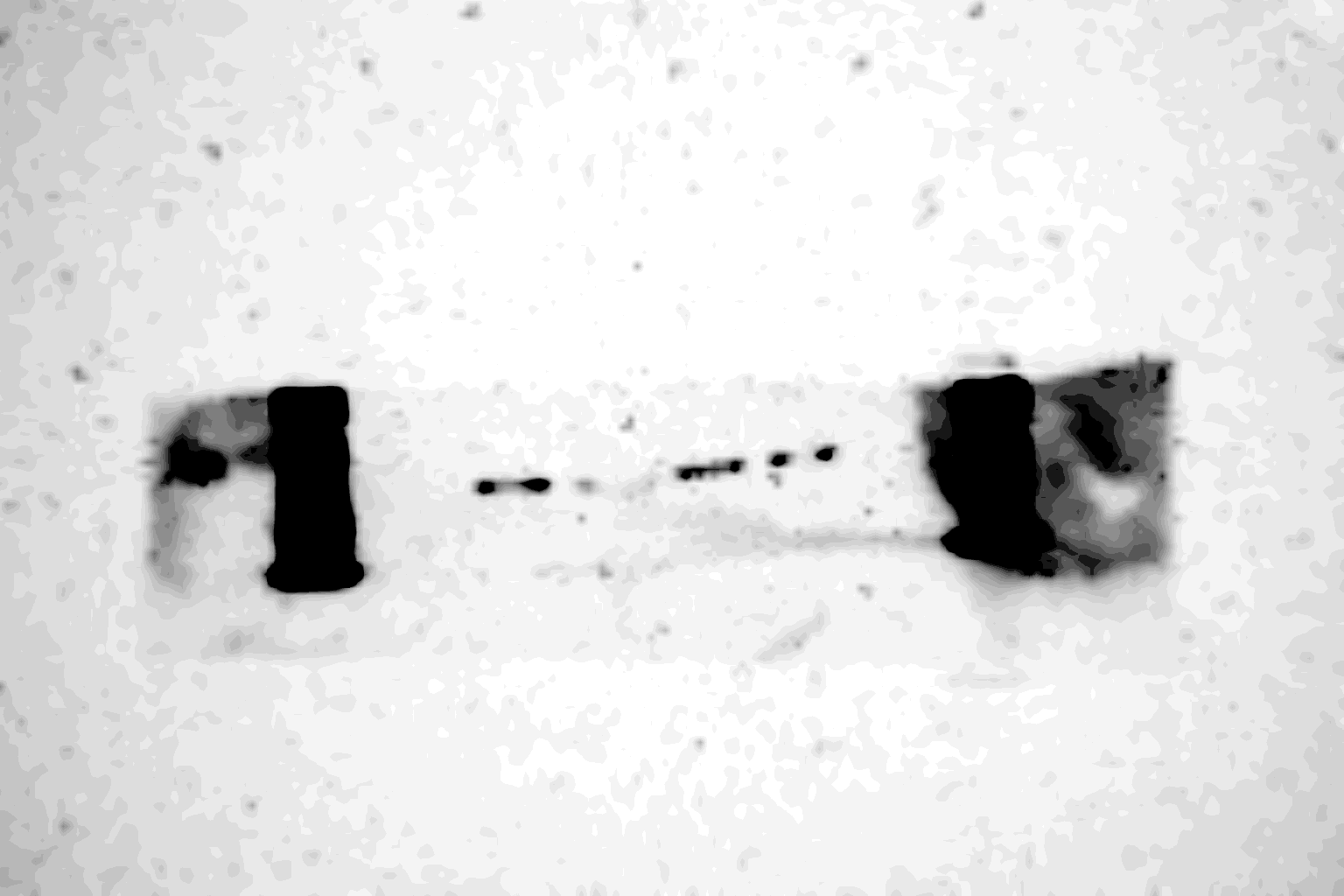

Supplement: Figure 4—source data 2. [file elife-106616-fig4-data2.zip › Figure 4_Source Data 2/╬▓-actin WB_20230328_1515.tif]
