# Supplementary material for: The oocyte zinc transporter Slc39a10/Zip10 is a regulator of zinc sparks during fertilization in mice
Source: eLife. 2025 Dec 11;14:RP106616. doi: 10.7554/eLife.106616 (PMC12698087; doi:10.7554/eLife.106616)
Supplement: Figure 4—source data 1. [file elife-106616-fig4-data1.zip › Figure 4_Source Data 1.pdf]

ZP2

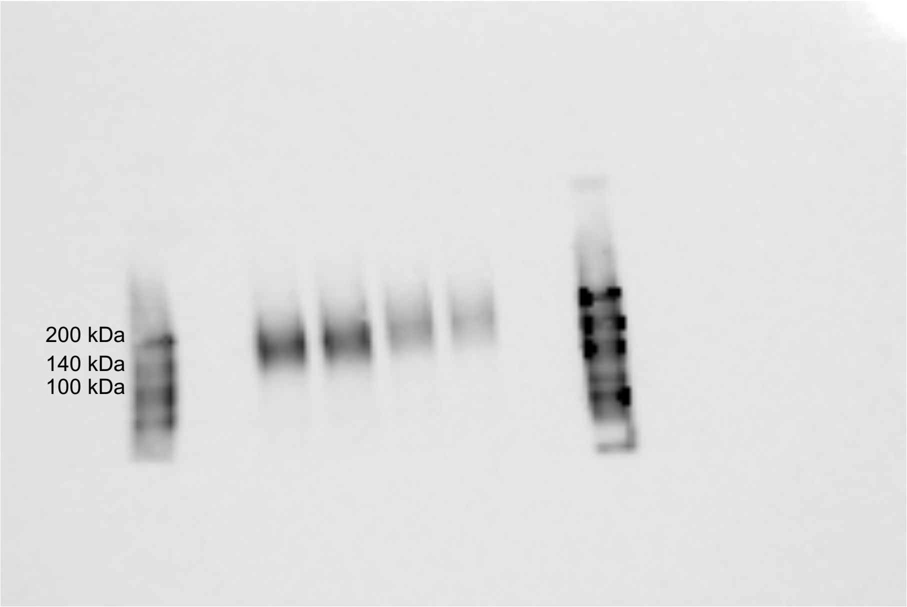

β-actin

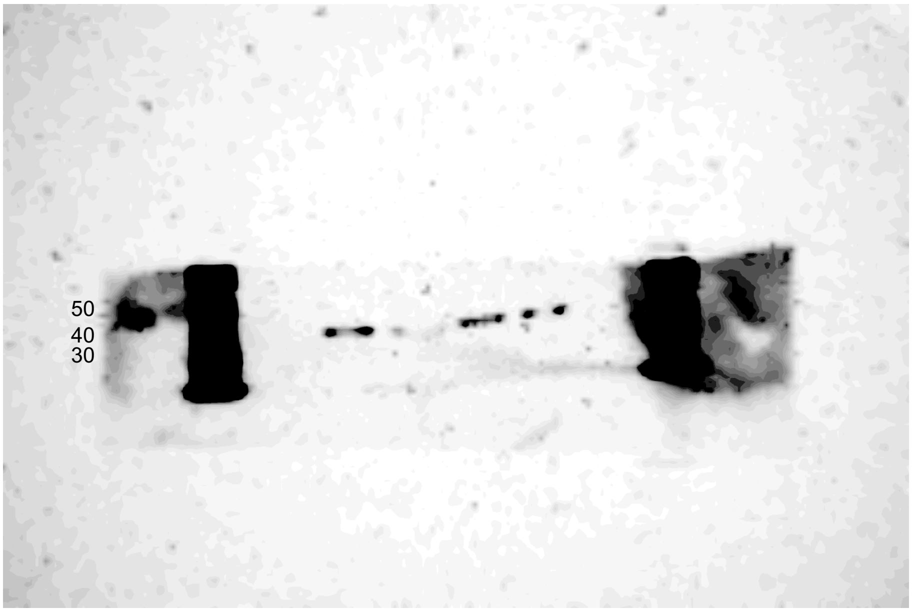

Figure 4-Source data 1. Original membranes corresponding to Figure 4D. Biotinylated Protein Ladder Detection Pack (#7727; Cell Signaling Technology) were employed as molecular weight marker. After blocking in WB, one membrane was cleaved at the 50-80 kDa position, with the upper membrane reacted with ZP antibody and the lower membrane reacted with β-actin antibody.
